# Supplementary material for: Attending physicians’ annual service volume and use of virtual end-of-life care: A population-based cohort study in Ontario, Canada
Source: PLoS One. 2024 Mar 8;19(3):e0299826. doi: 10.1371/journal.pone.0299826 (PMC10923452; doi:10.1371/journal.pone.0299826)
Supplement: S2 Table — (DOCX) [file pone.0299826.s003.docx]

**S2 Table. Baseline physician characteristics according to annual service volume before and during the pandemic**

| **Baseline Physician Characteristic** | **Annual Physician Practice Volume** | | | | | |
| --- | --- | --- | --- | --- | --- | --- |
|  | **Before the Pandemic** | | | **During the Pandemic** | | |
|  | Low (N=7,434) | Average (N=8,099) | High (N=2,802) | Low (N=7,876) | Average (N=7,221) | High (N=2,393) |
| Age (y), mean (SD) | 50.1 (13.3) | 50.0 (11.8) | 51.8 (10.6) | 51.0 (13.0) | 50.9 (11.5) | 52.3 (10.5) |
| Female sex, n (%) | 3,634 (48.9) | 2,883 (35.6) | 629 (22.4) | 3,746 (47.6) | 2,606 (36.1) | 566 (23.7) |
| Rural, n (%) | 502 (6.8) | 534 (6.6) | 55 (2.0) | 600 (7.6) | 384 (5.3) | 33 (1.4) |
| Canadian medical graduate, n (%) | 4,857 (65.3) | 4,947 (61.1) | 1,396 (49.8) | 4,935 (62.7) | 4,127 (57.2) | 1,127 (47.1) |
| Years in practice, median (IQR) | 22 (12-35) | 24 (13-34) | 27 (18-35) | 23 (12-36) | 25 (14-34) | 27 (19-35) |
| Practice specialty, n (%) |  |  |  |  |  |  |
| Family Medicine | 4,008 (53.9) | 5,151 (63.6) | 1,941 (69.3) | 4,458 (56.6) | 4,523 (62.6) | 1,643 (68.7) |
| Non-Cancer Specialist | 2,422 (32.6) | 1,714 (21.2) | 670 (23.9) | 2,407 (30.6) | 1,639 (22.7) | 568 (23.7) |
| Medical Oncologist | 207 (2.8) | 228 (2.8) | 39 (1.4) | 173 (2.2) | 254 (3.5) | 53 (2.2) |
| Surgical Oncologist | 745 (10.0) | 1,001 (12.4) | 151 (5.4) | 834 (10.6) | 805 (11.1) | 129 (5.4) |
| Status as a palliative care specialist, n (%) | 331 (4.5) | 289 (3.6) | 66 (2.4) | 352 (4.5) | 328 (4.5) | 97 (4.1) |
| Number of patients per physician  Median (IQR) | 3 (1-7) | 8 (4-15) | 11 (6-21) | 3 (1-6) | 7 (3-13) | 10 (5-19) |
| Number of visits in year prior to index  Median (IQR) | 2,221  (1,499-2,826) | 4,742  (4,001-5,691) | 9,678  (8,288-12,244) | 2,187  (1,450-2,784) | 4,627  (3,924-5,653) | 9,633  (8,255-12,095) |
| Number of end-of-life visits in year prior to index*  Median (IQR) | 0 (0-0) | 0 (0-2) | 0 (0-1) | 2 (0-7) | 7 (2-16) | 10 (3-22) |
| SD indicates standard deviation; IQR, interquartile range; MRP, most responsible physician | | | | | | |

**An end-of-life visit is defined by the presence of any of the following billings: 1) Home-based palliative care, 2) Virtual palliative care, 3) Any virtual visit billed with a home-based palliative care fee code in the last year of life, 4) Any virtual visit within the last 90 days of life*
