# Supplementary material for: Attending physicians’ annual service volume and use of virtual end-of-life care: A population-based cohort study in Ontario, Canada
Source: PLoS One. 2024 Mar 8;19(3):e0299826. doi: 10.1371/journal.pone.0299826 (PMC10923452; doi:10.1371/journal.pone.0299826)
Supplement: S3 Table — (DOCX) [file pone.0299826.s004.docx]

**S3 Table**. **Baseline characteristics of all community-dwelling adults in the last 90 days of life who died in Ontario according to their attending physician’s annual service volume before and during the pandemic.**

| **Baseline Patient Characteristic** | **Attending Physician Annual Practice Volume** | | | | | |
| --- | --- | --- | --- | --- | --- | --- |
|  | **Before the Pandemic** | | | **During the Pandemic** | | |
|  | Low (N=40,527) | Average (N=90,570) | High (N=45,230) | Low (N=37,299) | Average (N=67,498) | High (N=34,370) |
| Age (y), mean (SD) | 73.3 (15.7) | 75.4 (14.9) | 74.1 (16.2) | 74.1 (15.9) | 75.4 ± 15.1 | 74.0 (16.5) |
| Female sex, n (%) | 19,274 (47.6) | 41,270 (45.6) | 19,155 (42.4) | 17,515 (47.0) | 30,517 (45.2) | 14,470 (42.1) |
| Rural, n (%) | 6,352 (15.7) | 13,308 (14.7) | 3,445 (7.6) | 6,161 (16.5) |  | 2,415 (7.0) |
| Neighbourhood income quintile, n (%) |  |  |  |  |  |  |
| Lowest | 9,951 (24.6) | 22,212 (24.5) | 12,334 (27.3) | 9,549 (25.6) | 16,357 (24.2) | 9,274 (27.0) |
| Low | 8,689 (21.4) | 19,795 (21.9) | 10,312 (22.8) | 7,998 (21.4) | 14,623 (21.7) | 8,061 (23.5) |
| Middle | 7,704 (19.0) | 17,369 (19.2) | 8,916 (19.7) | 7,052 (18.9) | 13,092 (19.4) | 6,885 (20.0) |
| High | 6,758 (16.7) | 15,796 (17.4) | 7,185 (15.9) | 6,231 (16.7) | 11,784 (17.5) | 5,401 (15.7) |
| Highest | 7,225 (17.8) | 15,076 (16.6) | 6,325 (14.0) | 6,290 (16.9) | 11,358 (16.8) | 4,603 (13.4) |
| Missing | 200 (0.5) | 322 (0.4) | 158 (0.3) | 179 (0.5) | 284 (0.4) | 146 (0.4) |
| Surname ethnicity, n (%) |  |  |  |  |  |  |
| Chinese | 749 (1.8) | 1,917 (2.1) | 1,614 (3.6) | 711 (1.9) | 1,831 (2.7) | 1,289 (3.8) |
| South Asian | 464 (1.1) | 1,309 (1.4) | 1,700 (3.8) | 436 (1.2) | 1,187 (1.8) | 1,589 (4.6) |
| General | 39,314 (97.0) | 87,344 (96.4) | 41,916 (92.7) | 36,148 (96.9) | 64,470 (95.5) | 31,485 (91.6) |
| Chronic conditions, n (%) |  |  |  |  |  |  |
| Hypertension | 28,040 (69.2) | 67,374 (74.4) | 33,840 (74.8) | 20,840 (55.9) | 39,940 (59.2) | 20,840 (55.9) |
| Cancer | 27,417 (67.7) | 58,938 (65.1) | 26,181 (57.9) | 19,285 (51.7) | 35,241 (52.2) | 15,465 (45.0) |
| Diabetes | 13,614 (33.6) | 33,202 (36.7) | 17,890 (39.6) | 10,458 (28.0) | 20,283 (30.0) | 10,990 (32.0) |
| Heart failure | 9,922 (24.5) | 25,230 (27.9) | 13,106 (29.0) | 7,322 (19.6) | 14,359 (21.3) | 7,287 (21.2) |
| End-stage renal disease | 8,900 (22.0) | 21,801 (24.1) | 11,736 (25.9) | 6,797 (18.2) | 13,562 (20.1) | 7,267 (21.1) |
| COPD | 8,599 (21.2) | 20,030 (22.1) | 9,606 (21.2) | 5,889 (15.8) | 10,567 (15.7) | 4,961 (14.4) |
| Dementia | 5,877 (14.5) | 12,414 (13.7) | 6,155 (13.6) | 4,680 (12.5) | 8,048 (11.9) | 3,850 (11.2) |
| Stroke | 4,105 (10.1) | 9,804 (10.8) | 5,004 (11.1) | 3,189 (8.5) | 5,857 (8.7) | 2,853 (8.3) |
| Severe liver disease | 617 (1.5) | 1,144 (1.3) | 526 (1.2) | 573 (1.5) | 928 (1.4) | 359 (1.0) |
| Psychotic disorder | 976 (2.4) | 1,385 (1.5) | 904 (2.0) | 1,029 (2.8) | 1,125 (1.7) | 729 (2.1) |
| Nonpsychotic disorder | 10,261 (25.3) | 20,548 (22.7) | 11,168 (24.7) | 9,183 (24.6) | 15,859 (23.5) | 8,894 (25.9) |
| Alcohol and substance use disorder | 2,297 (5.7) | 4,086 (4.5) | 3,515 (7.8) | 2,349 (6.3) | 3,276 (4.9) | 3,022 (8.8) |
| Hospital frailty score |  |  |  |  |  |  |
| 0 | 5,000 (12.3) | 10,429 (11.5) | 4,694 (10.4) | 4,161 (11.2) | 7,493 (11.1) | 3,397 (9.9) |
| 0.1-4.9 | 9,748 (24.1) | 20,673 (22.8) | 9,695 (21.4) | 8,060 (21.6) | 14,688 (21.8) | 6,978 (20.3) |
| 5.0-8.9 | 4,928 (12.2) | 10,732 (11.8) | 5,162 (11.4) | 4,479 (12.0) | 8,010 (11.9) | 3,902 (11.4) |
| 9+ | 7,846 (19.4) | 17,584 (19.4) | 8,612 (19.0) | 7,395 (19.8) | 13,218 (19.6) | 6,612 (19.2) |
| LHIN |  |  |  |  |  |  |
| Central | 3,866 (9.5) | 8,637 (9.5) | 5,738 (12.7) | 3,430 (9.2) | 7,106 (10.5) | 4,572 (13.3) |
| Central East | 3,450 (8.5) | 10,050 (11.1) | 7,015 (15.5) | 3,455 (9.3) | 7,990 (11.8) | 5,100 (14.8) |
| Central West | 1,184 (2.9) | 3,559 (3.9) | 3,536 (7.8) | 1,092 (2.9) | 2,877 (4.3) | 3,340 (9.7) |
| Champlain | 5,874 (14.5) | 8,694 (9.6) | 2,248 (5.0) | 5,068 (13.6) | 6,137 (9.1) | 1,610 (4.7) |
| Erie St. Clair | 1,415 (3.5) | 5,042 (5.6) | 3,904 (8.6) | 1,349 (3.6) | 3,925 (5.8) | 2,905 (8.5) |
| Brant | 4,254 (10.5) | 11,375 (12.6) | 7,280 (16.1) | 3,622 (9.7) | 8,564 (12.7) | 5,250 (15.3) |
| Mississauga Halton | 2,006 (4.9) | 6,016 (6.6) | 3,313 (7.3) | 1,774 (4.8) | 4,656 (6.9) | 2,828 (8.2) |
| North East | 2,438 (6.0) | 5,317 (5.9) | 2,207 (4.9) | 2,511 (6.7) | 3,531 (5.2) | 1,585 (4.6) |
| North Simcoe Muskoka | 1,648 (4.1) | 4,783 (5.3) | 1,351 (3.0) | 1,665 (4.5) | 3,414 (5.1) | 1,040 (3.0) |
| North West | 1,232 (3.0) | 1,934 (2.1) | 767 (1.7) | 1,024 (2.7) | 1,356 (2.0) | 604 (1.8) |
| South East | 2,765 (6.8) | 4,627 (5.1) | 1,347 (3.0) | 2,619 (7.0) | 3,041 (4.5) | 859 (2.5) |
| South West | 3,690 (9.1) | 8,677 (9.6) | 2,255 (5.0) | 3,449 (9.2) | 6,297 (9.3) | 1,503 (4.4) |
| Toronto Central | 4,636 (11.4) | 5,678 (6.3) | 3,086 (6.8) | 3,856 (10.3) | 4,513 (6.7) | 2,251 (6.5) |
| Waterloo Wellington | 2,069 (5.1) | 6,181 (6.8) | 1,183 (2.6) | 2,385 (6.4) | 4,091 (6.1) | 923 (2.7) |
| Number of unique ED visits in past year not resulting in hospitalization, median (IQR) | 1 (0-2) | 1 (0-2) | 0 (0-2) | 0 (0-1) | 0 (0-1) | 0 (0-1) |
| Number of unique hospitalizations in prior year, median (IQR) | 0 (0-1) | 0 (0-1) | 0 (0-1) | 0 (0-1) | 0 (0-1) | 0 (0-1) |
| Receipt of any palliative care in prior year, n (%) | 3,985 (9.8) | 5,809 (6.4) | 2,124 (4.7) | 2,912 (7.8) | 4,303 (6.4) | 1,716 (5.0) |
| Receiving home care services in prior 2 years, n (%) | 16,923 (41.8) | 36,734 (40.6) | 17,228 (38.1) | 14,961 (40.1) | 26,985 (40.0) | 13,092 (38.1) |
| MRP, most responsible physician; SD, standard deviation; LHIN, Local Health Integration Networks; ED, Emergency Department; IQR, interquartile range | | | | | | |
