# Supplementary material for: Attending physicians’ annual service volume and use of virtual end-of-life care: A population-based cohort study in Ontario, Canada
Source: PLoS One. 2024 Mar 8;19(3):e0299826. doi: 10.1371/journal.pone.0299826 (PMC10923452; doi:10.1371/journal.pone.0299826)
Supplement: S4 Table — (DOCX) [file pone.0299826.s005.docx]

**S4 Table. Delivery of virtual and non-virtual care by a person’s attending physician in the last 90 days of life according to annual physician service volume, before and during the pandemic**

| **Care Delivered by a Patient’s Attending Physician** | **Attending Physician Annual Service Volume** | | | | | |
| --- | --- | --- | --- | --- | --- | --- |
|  | **Before the Pandemic** | | | **During the Pandemic** | | |
|  | Low (N=7,434) | Average (N=8,099) | High (N=2,802) | Low (N=7,876) | Average (N=7,221) | High (N=2,393) |
| Unique virtual EOLC visits, mean (SD) | 0.06 (0.3) | 0.07 (0.3) | 0.15 (0.6) | 1.14 (1.7) | 1.5 (1.4) | 1.83 (1.7) |
| Unique EOLC visits (in person or virtual), mean (SD) | 1.92 (1.8) | 1.92 (1.4) | 2.22 (1.5) | 2.18 (2.2) | 2.56 (1.8) | 3.08 (2.2) |
| Proportion of virtual visits to total unique EOLC visits, % | 3.1 | 3.6 | 6.8 | 52.3 | 58.6 | 59.4 |
